# Why monkeys do not get multiple sclerosis (spontaneously): An evolutionary approach

**DOI:** 10.1093/emph/eoy002

**Published:** 2018-01-23

**Authors:** Riley M Bove

**Affiliations:** Department of Neurology, UCSF, San Francisco, CA, USA

**Keywords:** evolution, multiple sclerosis, myelin, brain, hormone

## Abstract

The goal of this review is to apply an evolutionary lens to understanding the origins of multiple sclerosis (MS), integrating three broad observations. First, only humans are known to develop MS spontaneously. Second, humans have evolved large brains, with characteristically large amounts of metabolically costly myelin. This myelin is generated over long periods of neurologic development—and peak MS onset coincides with the end of myelination. Third, over the past century there has been a disproportionate increase in the rate of MS in young women of childbearing age, paralleling increasing westernization and urbanization, indicating sexually specific susceptibility in response to changing exposures. From these three observations about MS, a life history approach leads us to hypothesize that MS arises in humans from disruption of the normal homeostatic mechanisms of myelin production and maintenance, during our uniquely long myelination period. This review will highlight under-explored areas of homeostasis in brain development, that are likely to shed new light on the origins of MS and to raise further questions about the interactions between our ancestral genes and modern environments.

## INTRODUCTION

Multiple sclerosis (MS) is a complex neurologic disorder that is a leading cause of non-traumatic disability in young adults. Over the past generation, there has been a remarkable increase in the number of therapies targeting the immune dysregulation that leads to myelin destruction in MS. However, despite the plethora of contributing genetic and environmental factors identified [1], the underlying cause of MS remains unknown.

The goal of this review is to apply an evolutionary lens to understanding the origins of MS. This approach echoes that deployed in Robert Sapolsky's now classic examination of modern human stress and anxiety, “Why zebras don't get ulcers”. Here, we integrate three broad observations [2]. First, only humans are known to develop MS spontaneously. Second, humans have evolved large brains, with uniquely large amounts of metabolically costly myelin. This myelin is generated over long periods of neurologic development—and peak MS onset coincides with the end of ‘myelination’. Third, over the past century there has been a disproportionate increase in the rate of MS in women of childbearing age [3], paralleling increasing westernization and urbanization [4], suggesting sexually specific susceptibility in response to changing exposures.

From these three observations about MS, a life history approach leads us to hypothesize that MS arises in humans as a result of disruption, during our uniquely long myelination period, of the normal homeostatic mechanisms of myelin production, maintenance and clearance. Given the potential breadth of this topic, this review will focus on highlighting under-explored areas of homeostasis in brain development, that are likely to shed new light on the origins of MS and to raise further questions about the interactions between our ancestral genes and modern environments.

## BACKGROUND

### Multiple sclerosis

Multiple sclerosis (MS) is a complex neurologic disorder thought to affect ∼2.3 million individuals worldwide, with increasing prevalence [5]. It is characterized by episodic inflammatory attacks on myelin in the central nervous system (CNS), i.e. inflammatory demyelination. Eventually, the demyelinated axons undergo degeneration, leading to progression of symptoms and of disability. There is marked variability in the severity and extent of neurologic impairment that arises from MS lesions, including both visible (ambulatory or visual impairment) and less visible (fatigue and depression) symptoms; in the extent of recovery after inflammatory events; and in the pace of axonal loss and neurodegeneration. MS symptoms typically first develop during the childbearing years, but can begin before age 18 years in ∼5% [6], and after age 50 years in 10%, of individuals [7]. Consequently, MS is a leading cause of non-traumatic disability in young adults. Given the fact that many brain lesions may be ‘clinically silent’, i.e. do not cause classical MS relapse symptoms, there is sometimes a ‘prodromal phase’ during which lesions develop in the CNS without causing symptoms. This is supported by reports of increased healthcare utilization for all causes up to 5 years before an MS diagnosis [8], autopsy studies where in up to 25% of cases of pathologic MS, the patient had no diagnosis [9]; and studies of brain MRIs showing incidental, classic MS lesions in asymptomatic individuals (i.e. radiologically isolated syndrome) [10] who may or may not later go on to develop MS symptoms. Currently, all of the therapies approved for the treatment of MS are aimed at decreasing the number of inflammatory events (new lesions and consequent new clinical attacks, or relapses); none has a primary mechanism of preventing axonal degeneration.

MS is considered a complex genetic disease, with over 200 risk alleles identified; the most important of these, the human leukocyte antigen (HLA)-DRB15*01 allele, confers an odds ratio of 3.08 [11]. However, none is causative, and concordance among monozygotic twins is <30%, suggesting an important role for environmental factors [12–14]. Historically considered a disease affecting Caucasians living in northern latitudes, MS appears to be increasing among non-Caucasians in western countries, including children of foreign-born parents [15]. It also appears to be increasing in many regions of the world [5], such as Iran [16]. While this increase is often explained in terms of westernization and changing exposures, it is also likely that unequal access to diagnostic tools, such as MRIs, in developing countries leads to inequities in MS ascertainment [17]. Observational studies have shown a strong effect of early residence on risk of MS, demonstrating an important role for experiential programming of MS risk. Immunologically, MS is thought to arise as a result of shift from immune tolerance to, to active immunity against, myelin (particularly myelin basic protein, MBP, a major component of the myelin sheath), through activation of myelin-reactive lymphocytes. Allelic variants of HLA genes appear to shift T- and B-cell responses against myelin. The many environmental risk factors identified (all of which are commonly found in people without MS) include low vitamin D levels, exposure to Epstein-Barr virus (EBV) during childhood [18], and more recently, gut microbiota [19, 20]. Infectious exposures appear to stimulate pattern-recognition receptors on dendritic cells present in lymph nodes, potentially activating or re-programming T cells that circulate through the lymph nodes with myelin reactivity. Despite this plethora of contributing genetic and environmental factors identified [1], the underlying cause of MS remains unknown.

### Relevant evolutionary approaches

#### Life history theory

The evolutionary concept of life history theory provides a method to examine the evolutionary drives underlying the physiologic events occurring over an organism’s life, from fetal origins through senescence. At each step of development, an energy allocation tradeoff occurs between the various physiologic systems (e.g. [21–23]), with homeostasis altered by a range of environmental, social or other cues. To provide a classical example of these time allocation trade-offs, the tasks of somatic and cognitive development are delayed if a young child is fighting parasitic infections [24, 25]. The evolution of the long post-reproductive lifespan in humans, a feature shared only with some whales species, has also been examined through a life history lens [26]. According to the classical Grandmother Hypothesis [27], down-regulation of direct reproductive function and long post-reproductive life, may allow the organism to shift energy toward less direct reproductive contributions (in the form of parental or grandparental care to ensure the growth of highly dependent children, thereby ensuring that one’s offspring will successfully reproduce). A modification of this hypothesis, the Embodied Capital Hypothesis [28], posits that the longevity is advantageous to both men and women, who over the lifespan develop more successful brains and tools, allowing them to continue to acquire more resources than needed well into old age. However, with age it becomes more advantageous to reallocate these resources away from increasingly risky direct reproduction, and toward optimizing the well-being and fertility of children and grandchildren. Within neuroscience, the framework of ‘critical periods of plasticity’ [29, 30] is beginning to shed light on some of the specific developmental and homeostatic functions (guide, prune, refine and phagocytose) of glial cells to optimize neuronal development and function over the life span, which will enable more mechanistic investigations of life history tradeoffs.

#### Evolutionary mismatch

The concept of evolutionary mismatch [31] can be defined as ‘deviations in the environment that render biologic traits unable, or impaired in their ability, to produce their selected effects’ [32]. A classical illustration of the evolutionary mismatch theory is the Hygiene Hypothesis (that purports that modern hygienic practices alter development of the immune system by reducing its exposure to pathogens) [33]. The Old Friends Hypothesis, a more recent modification, attributes the increase in inflammatory chronic diseases to developing system's deprivation of stimulation from specific antigens, symbionts, helminths and other micro-organisms that were present over [34–36]. Evolutionary mismatch is also cited when examining the rise of non-communicable conditions such as obesity and cardiovascular disease. Developing populations are particularly vulnerable as they transition more rapidly than did western populations from adverse (hunger, infections, insecurity) to affluent (abundance, hygiene, security) environments, because of genetic and persistent epigenetic programming toward ‘thriftiness’ (i.e. storing excess calories), and delayed shifts in cultural ideals emphasizing slimness and activity [37].

### Three observations about MS

#### Observation 1. Only humans appear to develop MS spontaneously

There are to date no known cases of spontaneously occurring MS in other species. For example dogs, animals that live in the same households and are exposed to many of the same environmental factors as humans, are known to develop a number of autoimmune conditions, such as autoimmune thyroiditis [38], hepatitis [39], hemolytic anemia [40] and myasthenia gravis [41]. They also spontaneously develop a number of degenerative CNS conditions, many of which share hallmarks with their human counterparts; but not MS [42–44]. Similarly, we found no reports of MS-like disease in elephants, that have hippocampal volumes similar to humans’ [45], or in large-brained whales.

In humans’ closest living genetic relatives, the non-human primates, a demyelinating neurodegenerative process can be induced in response to viral and other antigen exposures. For example, the most common animal model of MS (experimental autoimmune encephalomyelitis) can be induced via injection of myelin proteins or T cells specifically reactive to these antigens, in rhesus and common marmoset monkeys. While a spontaneous MS-like disease, Japanese Macaque Encephalomyelitis, has been reported, it is linked to exposure to a previously undescribed, gamma-2 herpesvirus [46]. In the great apes, spontaneous MS has not been described [47]—and although it is possible that rare occurrences go unobserved in the wild, this is unlikely.

#### Observation 2. Humans experience a characteristic prolonged period of myelination

Myelination evolved in jawed vertebrates to improve the speed and efficiency of conduction of action potentials along long axonal distances. The existence of insulating myelin around most of the axonal length means that membrane potential-sensing ion channels can be aggregated at nodes where ion exchange is concentrated and focused, allowing saltatory, rather than membrane, conduction. This dramatically speeds action potential transmission, and is energetically favorable for the neuron. Myelin in the CNS is formed by oligodendrocytes (which differentiate from oligodendrocyte precursor cells, or OPCs), where it serves several other functions: providing physical and metabolic support for axons [48, 49] and possibly fine-tuning the exquisitely coordinated integration of action potentials coming in from multiple areas of the CNS. [50] Myelin, by increasing the conduction velocity of axons permits increased body size, rapid movement and a large and complex brain [51].

Relative to the non-human primates, humans have larger brains, resulting in exceptionally high basal metabolic rate and total energy expenditure [52], as well as greater white matter volume [53]. Humans’ unique long period of post-natal altriciality [54, 55] (slow childhood growth and reliance on others during development) was typically considered an accommodation of the ‘obstetrical dilemma’, i.e. between evolutionarily adaptive requirements for social and communicative brains and human bipedalism (wider birth canal and hips impose stress on bipedal females’ knees) [56]. More recently, parturition has been hypothesized to occur when the energetic requirements of the developing infant surpass the supply of the mother [57], and may coincide with the high metabolic demand of myelinating cells on neurons (e.g. lactate/glucose metabolism and transport from neurons to oligodendrocytes [48, 58]. Therefore, human infants fit through the birth canal with a limbic system, and then exhibit ongoing brain growth during their extended period of altriciality, to become the highly social animals capable of adapting to diverse environments.

Volumetric MRI studies are uncovering remarkably consistent spatial and temporal patterns of dynamic changes in human brain structure and maturation throughout childhood, adolescence, and young adulthood [59, 60]. It is generally accepted that cortical gray matter maturation of primary sensory and motor areas is followed by maturation of higher-order processing areas; and that white matter axonal maturation begins in central and caudal areas and progresses toward polar and rostral locations.

Astrocytes support the multiplication and differentiation of neurons, and often operate under sexually specific hormonal signaling [61]. This initial neuronal *multiplication* is followed by *pruning* of gray matter and synapses during *specification*, while oligodendrocytes myelinate the axons (forming white matter); and there appears a tight coordination between maturation of gray matter regions and their connected white matter tracts [62]. To support this development, microglia (resident macrophages in the CNS) identify infectious pathogens, plaques, and injured neurons; present them as antigens to T cells; and clear them through cytotoxicity, phagocytosis and synaptic stripping. Throughout development, then, while a highly coordinated series of peripheral immunologic events occurs, the developing CNS also requires constant immune homeostasis and surveillance, as well—in a manner that appears less ‘immune privileged’ than it is compartmentalized relative to peripheral blood [63].

Overall, human brains exhibit slow childhood growth curves, and then, over a period of years (and with a range of input from parents, siblings, grandparents and other helpers), prune and myelinate the developing neocortex through the post-pubertal period, until their brains reach peak brain size by early adulthood [64–66]. Both neuronal *progressive* (cell growth and maturation, myelination) and *regressive* (synaptic pruning, cell death and atrophy) processes are at work. Accompanying structural changes, functional imaging studies have revealed progressive maturation of task-specific networks important for the acquisition and enhancement of skills and behaviors [67], and for the development of higher cognitive function in the transition from childhood to mid-adulthood [68, 69]. Deviations from this developmental trajectory appear strongly related to altered cognitive performance [70] and neurologic impairments [71].

Burgeoning observations in rodents support the concept of critical windows of plasticity for myelin regulation. For example, OPC density is regulated through a balance of active growth and self-repulsion, ensuring that OPCs are available to replace oligodendrocytes and participate in tissue repair [72]. In mice, activity dependent plasticity is supported by data from both motor learning (leading to increased OPC differentiation [73]) and sensory deprivation (demonstrating dynamic modulation of myelin during critical periods of plasticity [74]) experiments. To support the metabolic requirements of OPC differentiation, OPC-intrinsic hypoxia-inducible factor signaling couples postnatal white matter angiogenesis, axon integrity, and the onset of myelination in mammalian forebrain [75]. As myelin sheaths age, they gradually release myelin pieces, which are subsequently cleared by microglia (forming insoluble, lipofuscin-like lysosomal inclusions) [76]. Importantly, due to differences in the relative duration of life history stages (e.g. prolonged development and duration of postreproductive lifespan in humans), windows of plasticity identified in rodents or other animal models may be unlikely to capture the range of windows in humans, limiting our understanding of this process in humans.

It is becoming increasingly clear that throughout the life course, other cells seem to actively support this plasticity: glial cells interact dynamically with neurons to guide, prune, repair and clear away debris from complex neural circuits. For example, microglia, which mature according to a stepwise developmental program, integrate tissue-specific immune response pathways in response to immune stimuli (e.g. gut microbiome or prenatal stress) [77], rapidly repopulate the brain after microglial depletion [78], and are now known to play a role in remodeling neural circuits [79, 80]. Furthermore, removal of waste plays an important role in brain development and homeostasis, with reduced waste clearance as individuals age noted for some time. Initially, clearance of 24S-hydroxycholesterol (primarily produced in the brain) was reported to fall markedly after age 20 [81]. To what extent this reflects decreased clearance, or decreased rates of production, of this marker is uncertain. The description of the paravascular glymphatic system in recent years, including its ability to be non-invasively visualized using MRIs [82], will dramatically expand the scope of inquiry into regulation of brain homeostasis and immune surveillance. This glymphatic system facilitates the flow of cerebrospinal fluid (CSF) and the clearance of interstitial solutes (including amyloid B) [83], and its efficiency is impaired in the aging brain [84].

**Figure 1.**
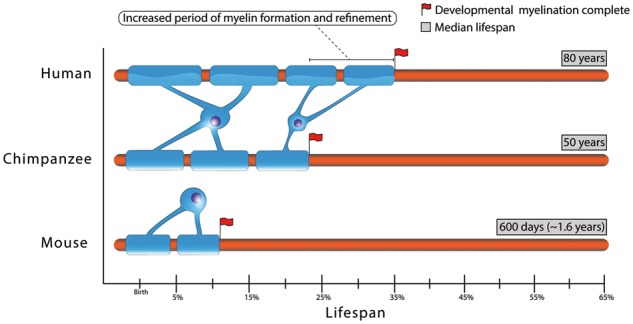
Schematic comparison of the period of developmental myelination relative to median lifespan in humans, chimpanzees and mice. The temporal demarcation provided represents the approximate period by which maximal developmental myelination has occurred, based on currently available data. Beyond this period, some ongoing myelination (including adaptive myelination) may continue to occur albeit at lower rates [88–92]

By comparison with human’s closest living phylogenetic relatives, chimpanzees, human myelination appears clearly prolonged (Fig. 1). This is also true when compared with the highly sophisticated brains of dolphins, which feature advanced myelination at birth [85]. In fact, neuropathologic and MRI studies have revealed distinct developmental schedules of neocortical myelination between the two species: at birth, chimpanzees have more myelinated axons than humans, and myelin growth appears complete around the time of reproductive maturity; whereas humans experience a period of post-pubertal myelin growth that continues into the third decade, developing in primary cortical areas before association areas [64]. Further, heritability for brain size and cortical organization appears to be lower in humans, particularly in association areas [86], and there is a delay in the prefrontal cortex gene expression profiles in humans [87]. These observations suggest a species-specific neuroplasticity inviting an increased role for environmental (ecological, social, cultural) factors in stimulating activity-dependent myelination during development. As the periods of adolescence and young adulthood appear quite novel in human evolution, the functional significance of post-pubertal neurologic maturation could be in promoting additional gains in cognitive skills relating to executive function. However, disruption in homeostatic regulation of this protracted and plastic process of myelination could also be a factor leading to neuropsychiatric diseases such as schizophrenia [93], and MS. Interestingly, the peak age of MS onset (age 24 in women and 25 in men [94]), i.e. of individual vulnerability to CNS inflammatory events, coincides with the time around which this myelination period is typically considered complete (some myelination in frontal and temporal lobes may continue until the fifth decade [95]), i.e. when the work of oligodendrocyte activity slows.

#### Observation 3. Life histories are sex-specific, and humans worldwide are experiencing rapidly changing sex-specific epidemiologic exposures

A central aspect of human reproductive physiology is responsiveness to cues regarding the optimal physiologic and environmental conditions to sustain a pregnancy/father a child, and then raise this child over decades. In addition to sex chromosome effects, sex hormones appear to regulate a number of aspects related to myelin production and regulation. Responsiveness of the female immune system to gonadal steroids (including those during menstrual cycling) is evolutionarily adaptive, enabling the immunotolerant state of pregnancy [96, 97]. In the CNS, there is evidence of hormone-, task- and region-specific menstrual cycle phase-dependent neural plasticity [98–103] and modulation of gray matter volume [104]. With respect to myelin regulation, most notably modulation of the estrogen receptors (ERα and ERβ [105], and the membrane-associated G protein-coupled receptor 30, GPR30 [106, 107]) have been reported to induce remodeling of the oligodendrocyte cytoskeleton [108, 109], and to stimulate both endogenous myelination as well as remyelination after demyelination [110–112]. Androgens in contrast appear to attenuate the stimulation induced by myelin basic protein-primed T cells on astroglial activation and production of proinflammatory molecules (e.g. IL-6 and IL-1β) [113]. Finally, progesterone may modulate a number of inflammatory processes, ion and water homeostasis and myelin repair in the injured CNS [114–116].

Over the past century there has been a disproportionate increase in the rate of MS in young women of childbearing age, moving from near-equal ratios to a 3:1 female:male ratio [3]. Interestingly, women with MS are more likely to carry the HLA DRB1*1501 risk allele than are men, but otherwise have lower non-HLA genetic risks scores [117], indicating that interactions of HLA genes and novel exposures may account for some of the increased risk in women. In fact, the increase in the sex ratio parallels increasing westernization and urbanization [4], sexually specific plasticity in response to changing exposures, including industrialization, sedentarism, and effects of the demographic transition and gender equity on women’s age at menarche and reproductive choices (age at first birth, parity, breastfeeding and exogenous hormone use) [118]. These changing ecologic and sociologic influences regulating life history transitions may play an important role in altering homeostatic regulation of myelin and hence propensity for MS.

## THEORETICAL FRAMEWORK, INITIAL FINDINGS, AND PREDICTIONS

### Hypothesis and framework

A life history approach to integrating our three observations leads us to hypothesize, first, that given the absence of spontaneously occurring MS in other species despite the broad potential for mammals to develop autoimmune diseases, and given the specific potential of non-human primates to develop an MS-like demyelinating disease under stimulated conditions, then something specific about the pace or pattern of human myelination must shift the immune system toward reactivity against myelin. Second, the extended period of myelination of the human brain, evolutionarily adaptive in allowing a remarkable degree of plasticity in response to internal and external cues, may also provide an expanded time window during which homeostatic mechanisms of myelin production and maintenance become dysregulated as a result of perinatal or childhood exposures, resulting in enhanced myelin breakdown. As the rate of myelination slows in the third decade, there could be failure to downregulate the immune surveillance required for regulation of myelination and neuronal loss (both of which were critical to the developing brain), thereby shifting toward a pro-demyelinating state. This would increase the likelihood of local immune-driven demyelination [119], secondarily expanded and reinforced by the circulating innate immune system, in individuals with genetic susceptibility. Finally, in increasingly novel environments, the changing ecologic and sociologic influences that partially regulate life history transitions likely result in altered homeostatic regulation of myelin—which is highly sensitive to environmental modulation during development—and hence propensity for MS.

### Evidence and predictions across developmental transitions

To evaluate the influence of the protracted nature of human myelination on human susceptibility to MS, the evidence available to date from humans to directly support or refute these hypotheses remains scant, and may partially reflect the fact that animal models incompletely capture the range of variability that occurs during humans’ protracted myelination.

Further, given the limited opportunities to conduct neuropathologic studies in the brains of healthy young people, new tools are needed. Among these non-invasive tools, the fields of neuroimaging and epigenetics hold promise. Non-invasive neuroimaging techniques that allow for dynamic visualization of glial cell biology, including OPC activity and myelin formation are being optimized. Such techniques include magnetization transfer imaging ([120, 121], including magnetization transfer ratio [122], diffusion-weighted imaging and T2 relaxometry [123–125]; see [126] for a review], MR spectroscopy [127], and positron emission tomography (PET [128, 129]). Other neuroimaging tools are advancing the field of ‘connectomics’ [62, 130, 131], allowing a more precise evaluation of the spatial and temporal connection of CNS tracts and regions across development. The field of epigenetic regulation of MS is still-nascent [127, 128]. Initial epigenetic signals include sex biases in MS transmissibility and in genetic risk (women more likely to carry the HLA DRB1*1501 MS risk allele, but men with higher non-HLA MS genetic risk burdens [117, 132]). This suggests sex-specific interactions with MHC risk alleles, including epigenetic modifications [133], which remain sparsely explored [134]. As epigenetic insights unfold, so will our mechanistic understanding of how differential exposures during development increase susceptibility to MS.

To close this gap in understanding as new tools come online, application of evolutionary concepts—life history and evolutionary mismatch—holds significant potential to generate new, testable hypotheses likely to advance research on opportunities for modulation of myelin refinement and MS risk during humans’ protracted myelination.

#### Prenatal environment and early childhood

If MS risk is influenced by developmental exposures, then the prenatal and perinatal periods should play a particularly large role in shaping more proximal myelin development, but also in programming lifelong proinflammatory trajectories and myelin regulation. For example, prenatal hormone levels have been associated with MS risk [135, 136] and most convincingly, low prenatal levels of the hormone vitamin D [137], which may set the stage for development and intergenerational immunologic changes [138, 139]. Similarly, maternal adiposity, whose effects on offspring immune trajectories could result from metabolic programming of growth trajectories or the timing of puberty, is also beginning to show an association with MS [140]. Among possible contributors to myelin regulation (e.g. prenatal stressors [141] and specific toxins [142, 143]), the synthetic xenoestrogen bisphenol-A (BPA), has been reported to impair proliferation and differentiation of OPCs in the prenatal and postnatal periods as well as decrease the expression of genes regulating myelination [143]. Work is ongoing to understand the role of these exposures, and the effect of their early or late manipulation (e.g. through antibiotics, diet and transfers for the gut microbiome; supplementation for vitamin D levels), on myelin and immune regulation.

#### Childhood

After birth, neurons continue to migrate from the ventricles along a migratory ‘Arc’ to cortical regions, differentiating into more mature populations, such as inhibitory interneurons in the infant orbitofrontal cortex [144]. Cortical gray matter volume peaks around age 4 years, declining thereafter. Yet as a result of ongoing myelination, cortical white matter volume increases rapidly until age 10, with steady continued development until early adulthood (about age 20 years). In fact, total intracranial volume increases by about 300 ml from 3 months to 10 years. There is a wave of brain growth during childhood and adolescence: around 9 years of age the rate of annual brain growth is 1% until at age 13 years, when a gradual volume decrease sets in. In young adolescence, close spatial relationships have been observed between gray matter density reduction in frontal and anterior regions, and total brain growth. Together, they determine the ultimate density of mature frontal lobe cortical gray matter [145]. Subcortical structures in contrast do not show consistent changes [146].

During childhood, evolutionary mismatch has particularly important sequelae. As postulated in the previously referenced Hygiene [33] and Old Friends Hypotheses [32–34], exposure to an overly sanitized environment [35, 147–156] that lacks certain pathogens, appears to influence downstream immune function [157–167] as well as microglial activation, cholinergic development and inflammatory responses in the developing brain [168–170]. ‘Modern’ exposures, such as high-salt diets and eventual use of antihypertensives, could also influence other aspects of myelin homeostasis including regulation of waste removal from the CNS (reviewed in [171]).

Indeed, MS risk has been linked with exposure to a number of environmental stimuli such as obesogenic industrialized environments ([172–177], potentially mediated by leptin [162, 163, 178–181]), dietary factors such animal protein [182] (salt intake, while investigated, no longer appears to be a prominent risk factor [183]), and toxins [136, 184, 185]. Of particular interest is the gut microbiome, a major point of contact between the immune system and novel antigens during development [184, 186–199], with consequences for brain development [200], including CNS microglial function [20, 201, 202]. With respect to myelin refinement, commensal gut flora may stimulate various lineages of the immune repertoire, including myelin-specific CD4(+) T-cell responses [203] and autoantibody-producing B cells, in the presence of the target autoantigen, myelin oligodendrocyte glycoprotein (MOG) [204]. Supporting a regulatory function of diet and gut microbiome on myelin formation, in germ-free mice, the presence of hypermyelinated axons in the prefrontal cortex has been reported; these changes are potentially reversed by colonization with a conventional microbiota [205]. Similarly, depletion (through antibiotics) and transfer of gut microbiota in mice results in changes in prefrontal cortex gene expression and myelination [206]. Interestingly, associations between adiposity [174, 175], or gut microbiome [192, 207, 208] and autoimmunity appear more pronounced in girls, opening the door for comparative studies that would reveal mechanisms for immune responsiveness to these cues.

#### Adrenarche and puberty

Critical for regulation of immune, metabolic and reproductive physiology by a number of exposures [210–212], the pubertal transition corresponds to an apparent female-specific increase in MS risk [213]. Earlier puberty represents a risk factor for MS [214], and for earlier onset of MS symptoms [215, 216] in girls, but not so clearly in boys [213], which remains an area of active study [217]. Here, puberty may represent a proxy for earlier risk exposures that influence both downstream immune signaling and pubertal age (such as body weight [218–224] or shared genes [225, 226]), perhaps in response to hormonal signaling from leptin, amongst other hormones [218, 221, 227]. Interestingly, the developmental genetic susceptibility to elevated BMI, which increases MS risk [228], may have only been ‘uncovered’ in the more obesogenic environments of the recent past [229].

But hormonal changes that occur at puberty also influence immune pathways [224, 230] and thereby immune activity [231]. In fact, an emerging body of work supports an inflection point in a number of maturational changes in the associative cortex, including of gray matter, white matter, interneurons, synapses, neurotransmitter expression, among others—substantial direct evidence is needed to support hormonal regulation of maturation (including myelination) (reviewed recently in [232]).

With respect to hormonal cycling, the greater number and regular frequency of periods that women experience in westernized societies relative to women in other societies or throughout our evolutionary past (e.g. [233, 234]) could provide more frequent opportunity for altered (*dys*)regulation of these immune and neurologic responses, contributing to a pro-inflammatory MS state. Here, extended-cycle hormonal contraceptives (i.e. cycles every 3 months) might, in comparison with typical hormonal contraceptives (i.e. monthly cycles), be hypothesized to result in fewer demyelinating inflammatory triggers (fluctuations in pro-inflammatory cells) and fewer new MS lesions.

#### Late adolescence and young adulthood

During young adulthood, between ∼18 and 35 years of age, possibly another wave of growth, or at least a period of no brain tissue loss, occurs [60]. As stated above, the peak age of MS onset, i.e. of individual vulnerability to CNS inflammatory events, is in the mid-20s, perhaps slightly earlier in women than men [94]. This peak age of individual vulnerability to CNS inflammatory demyelination coincides with the time that myelination is considered complete, i.e. when the work of oligodendrocyte activity slows.

To return to our hypothesis, we expect, first, that a background process of myelination and remyelination will be found to be dynamic in healthy brains, particularly during childhood and adolescence. The concept of experience-dependent myelination in response to a new activity or skill acquisition, hypothesized to occur in an early study demonstrating changes in the auditory cortex after cochlear implants [235], has been bolstered by evidence of tract changes in neuroimaging studies of children and adolescents practicing piano [236], and of young adults acquiring a visuomotor skill [237]. Conversely, selective loss of myelin would be expected to occur in a tract whose gray matter was injured from a vascular or accidental event.

Second, as the pace of myelination slows in young adulthood, the local CNS immune regulation, both innate and adaptive, should be expected to downshift myelin modeling, including by decreasing immune cells targeting myelin. Therefore, during late adolescence and young adulthood, a greater number of glial and immune cells would be expected in late-myelinating tracts relative to regions with more complete myelination, and overall in late adolescence relative to early adulthood.

Third, MS would then arise in individuals who, due to ‘multiple hits’ (which influence immune activation or myelination) incurred during the protracted period of myelination, are less successful at shifting from a dynamic state of myelin remodeling in adolescence (with active immune surveillance, myelin formation and debris clearance) to a less dynamic homeostatic balance in adulthood, resulting in ongoing and more targeted attacks on myelin. There are some signals that B cells specific to the myelin surface antigen MOG might, through antigen-presenting cells, activate MOG-specific effector T cells either within the CNS or peripherally [238]; and that there is age-dependent autoimmunity to MOG in MS [239]. However, the significance of MOG as an antigen or a bystander remains unclear [240]. After an initial demyelinating attack, some patients do seem more able to achieve CD4(+) T-cell quiescence [241], avoiding further demyelination. However, more mechanistic insights are needed to understand how immune surveillance and glial activity change, and can become pathological, once the rate of myelination slows.

It remains to be proven whether the tracts that are latest to myelinate are the first to experience demyelination. While a number of studies have reported the general anatomical location of first clinical attacks (i.e. optic nerve, spinal cord, brainstem, e.g. [242]), ascertainment of this earliest stage is limited by the high number of MS demyelinated lesions that are asymptomatic (several lesions may be present at the time of the first clinical attack). Even when demyelinated lesions are incidentally noted on an MRI obtained for other purposes (radiologically isolated syndrome [10]), the requirement that these lesions be spatially disseminated across the CNS to fulfill criteria for demyelinating disease, may limit ascertainment of the location of the very earliest lesion. Certainly, it has been shown that spinal cord lesions strongly predict recurrent demyelination [243, 244], but it is not clear whether this is biologically meaningful (i.e. that spinal cord lesions signal or result in increased immune activation) or whether they simply provide more specificity in distinguishing demyelinating lesions from the other non-demyelinating causes of white matter abnormalities in the brain. The pattern of *re*myelination in humans with MS, as well as of remyelination after toxic injury in mice, does appear to be influenced by neuroanatomical location (e.g. may be faster in the murine corpus callosum than in the cortical gray matter [245]; and more extensive in subcortical lesions than periventricular lesions and near absent in cerebellar lesions in humans [246]).

As the epigenetic signatures that mark stages of brain development [61, 247–249], including completion of myelination, as well as proinflammatory profiles in circulating blood and the CNS, are identified, it will become possible to more precisely query the temporal relationship between myelination and the activated immune system, and to identify potentially aberrant patterns in people with underlying genetic susceptibility to MS.

### Life history approaches beyond development

#### Aging in MS

Beyond raising important questions about MS risk, evolutionary approaches continue to be informative in examining other aspects of MS, such as the process of accelerated aging. In all individuals, after age 35 years, a steady volume loss is found of 0.2% per year, which accelerates gradually to an annual brain volume loss of 0.5% at age 60. This loss appears driven primarily by curvilinear declines in cortical gray matter volume, while cortical white matter volume remains constant during the first five decades [250–252], but then may decrease (in mice). This process of brain volume loss is accelerated in individuals with MS. Before the 6th decade, i.e. broadly the age of menopause and declining ovarian function, women appear to have a relatively slower rate of disability accrual than men despite higher risk of relapses [253]. More research is required to determine whether this is due to gonadal hormone effects on remyelinating capabilities [109] during this time (possibly to enable pregnancy-induced brain plasticity) or other mechanisms altogether.

Aging is associated with a number of impairments of remyelination, including decreased OPC recruitment and differentiation [254, 255], and shorter internodes [256–258]. These age-associated changes likely arise from changes in environmental signaling [259] and in epigenetic regulation [260, 261]. The effect of increasing age on impaired remyelination is further supported by reversal of age-associated remyelination deficits with telomerase reactivation [262] and with enhancement of remyelination after heterochronic parabiosis, in which unidentified circulating factors may respectively inhibit or enhance remyelination including blood-derived monocytes from a younger mouse recruited to demyelinating lesions, by increasing M2 microglia and macrophages in these lesions [263, 264]. In humans, OPCs may represent a smaller proportion of the total glial population [265] than in other mammals such as rodents, where they make up ∼8–9% of the total cell population in white matter [266].

Once damaged, myelin debris may prematurely shift microglia toward aging, rather than earlier, immune regulation pathways, leading to premature brain senescence [77]. Some support for hypothesis derives from a still unreplicated report of that mutations in the LXRA gene may be presents in some cases of familial progressive MS [267]; these mutations are postulated to decrease innate immune suppression of pro-inflammatory mediators, leading to exaggerated attack on myelin as well as impaired remyelination, accelerating axonal loss and MS progression [267].

#### Female-specific exposures: reproductive events

**Figure 2.**
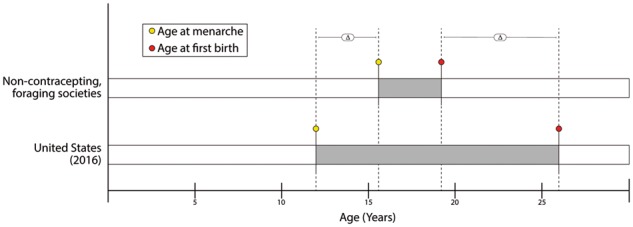
Schematic comparison of the period from menarche to first birth in U.S.-born girls relative to non-contracepting populations. U.S. birth and menarche data were obtained from [274, 275]. For comparison purposes, data were averaged from 7 communities whose reproductive histories are considered more typical of those encountered over human history: Ache, Agata, Dogon, Hazda, Hiwi, !Kung, and Pygmy (East) [28, 276–280]

To understand this sex-specific influence on MS risk, here as well, the life history and evolutionary mismatch approaches may be informative. A major aspect of altered life history in modern decades is delayed age of childbearing, which combined with earlier menarche, translates to more years of exposure to hormonal cycling (Fig. 2). Both nulliparity and delayed pregnancy appear to increase the risk of MS [268, 269]. It is now well established that there is a dramatic decline in relapse risk of MS in the last trimester, with a rebound in relapses (for up to 30% women) noted in the first trimester postpartum [270, 271]. The increase in relapses postpartum coincides with dramatic hormonal shifts and loss of the immunotolerant state of pregnancy [272, 273]. A number of immune mechanisms have been postulated, including, classically, induced shifts from T-helper 1 to T-helper 2 responses [281], as well as fetal HLA-maternal killer immunoglobulin-like receptors interactions [282, 283], microchimerism derived from fetal cells or antigens [284, 285], and placenta-derived immunoregulation [286]. Hormonal signaling during breastfeeding, reflecting the metabolic demands placed on the mother [287, 288], may also play a role in modulating immune activity and relapse susceptibility in MS [289]. Both the increased years of exposure to hormonal fluctuations (from menarche to childbearing), as well as delayed or reduced exposure to immunotolerant state of pregnancy, could shift or maintain the immune system in a pro-inflammatory state, with a greater potential for activation against myelin than in women with less hormonal cycling or earlier pregnancies.

In addition to immune effects, pregnancy also induces brain plasticity [290], demonstrating tight integration of signaling along the hypothalamic–pituitary axis [291] to coordinate release of oxytocin and prolactin [292–294] and enable the physiology of gestation and lactation for a current, and even subsequent, pregnancies [295]. Beyond these physiologic hormonal adaptations, higher-level cognitive and affective processes also appear to change during pregnancy [296], possibly enabling broader preparations of the female brain for motherhood. These include for example changes in the expression of oxytocin receptors in brain regions beyond the hypothalamus and pituitary, to permit centrally released oxytocin to facilitate the expression of maternal behavior [297]; or decreased olfactory thresholds during the first trimester to potentially protect the mother and developing fetus from toxic ingestions [298, 299]. Most interestingly altered hippocampal neurogenesis and plasticity [300–302], as well as loss of gray matter volume in areas modulating social cognition [303], have been reported in gestation and the postpartum period, with unknown functional consequences but possible relevance to postpartum mood and memory disorders [304]. Prospective studies of young women during a pregnancy compared with nulliparous controls will be substantially enhanced with neuroimaging and epigenetic tools that query the influence of the timing of pregnancy-related neural and immunologic changes, relative to broader patterns of immunologic and brain maturation, and consequent immune activation against myelin.

## CONCLUSION

Humans have evolved a highly complex brain, and its maturation is both highly coordinated, and thanks to an extended period of myelination, demonstrates a remarkable degree of plasticity in response to internal and external cues. While one environmental cause for MS in humans may yet be identified, from an evolutionary lens, MS could represent an unfortunate byproduct of this sophisticated brain, specifically arising from a disruption in local homeostatic mechanism of myelin regulation during the protracted and plastic period of myelination and in response to a number of cumulative, and interactive, genetic and environmental risks. A similar perspective has been proposed for both neuropsychiatric disorders such as schizophrenia [93], and neurodegenerative disorders such as Alzheimer’s disease [304]. Just as in other species, these risks would have the most profound impact with exposure early during development, but would have a longer time frame in which to affect myelin regulation than in other species. Therefore, the incidence of MS is expected to continue to increase, and specifically in non-Caucasians due to evolutionary mismatch as they immigrate to regions of high MS prevalence, or as their native environments are increasingly westernized. One major scientific implication of this perspective is that animal models, which do not manifest this protracted period of myelination, inadequately unravel the extent of the interaction between risk factors, immune regulation, and glial biology during human development. In the coming years, the fields of epigenetics and advanced neuroimaging—which illuminates brain connectomics and glial biology—all promise to shed further light on the dynamic maintenance of homeostasis within the CNS across humans’ protracted development, and to answer the question of why only humans appear to spontaneously develop MS.
